# Co-designing solutions to tackle food insecurity in higher education settings: a scoping review

**DOI:** 10.1017/S1368980025000485

**Published:** 2025-04-14

**Authors:** Taylah Scutts, Nadia Farnaz, Gantsetseg Ganbold, Alexandra J Bhatti, Shirley Phan, Miriam J Williams, Seema Mihrshahi

**Affiliations:** 1 Department of Health Sciences, Faculty of Medicine, Health and Human Sciences, Macquarie University Wallumattagal Campus, Macquarie Park, NSW 2109, Australia; 2 School of Communication, Society and Culture, Macquarie University Wallumattagal Campus, Macquarie Park, NSW 2109, Australia; 3 Buildings and Property Division, Clayton Campus, Monash University, Clayton, VIC 3800, Australia

**Keywords:** Food insecurity, Higher education, Food access, College and university students, Co-designing

## Abstract

**Objective:**

Food insecurity (FI) in the higher education setting is a pressing social justice and public health nutrition issue. Persistent FI rates among students suggest that the current programmes and institutional policies are inadequate. Engaging the community in co-design practices can enhance research and decision-making, leading to more targeted advocacy and solutions. This review describes and evaluates evidence of co-design approaches and identifies strategies for addressing FI in higher education settings.

**Design:**

A review was conducted using the Preferred Reporting Items for Systematic Reviews and Meta-Analyses extension for Scoping Reviews. Literature was searched in three electronic databases (Scopus, Ovid MEDLINE and Web of Science) and two search engines (Google and Google Scholar).

**Setting:**

Only studies based in higher education settings were included.

**Participants:**

Higher education students.

**Results:**

The search identified 814 studies, of which twenty-eight met the inclusion criteria. Studies involving co-design and participatory research frameworks had higher participation, leading to increased student awareness of FI, student leadership and the development of campaigns and collaborative organisational structures. A content analysis approach identified seven categories for strategies targeting student FI: (1) policy and institutional support; (2) strategic partnerships (3) advocacy and awareness; (4) initiatives for student engagement; (5) student skills and knowledge development; (6) programme development and (7) campus food environment.

**Conclusions:**

Co-designed research methodologies are important for addressing student FI, enhancing advocacy and understanding stakeholder needs. Future studies should prioritise collaborative approaches when exploring solutions to FI and similar social justice issues affecting students.

Food insecurity (FI) among higher education students, hereafter referred to as students, is an ongoing public health nutrition issue. Recent studies conducted across higher education institutions have reported prevalence estimates ranging from 19 % to 56 %^(1–4)^. FI is defined as a lack of access to healthy, nutritious, culturally appropriate and affordable foods^(5)^. This issue has been increasingly studied in high-income countries, with contributing factors including the recent pandemic, cost of living pressures and increasing privatisation of campus food outlets in higher education settings^(6,7)^.

As a result, students are more likely to skip meals and compromise the nutritional quality of their food^(6)^. In recent years, this has led to a growth in research efforts to understand the complexity of student FI and develop solutions.

Commencing higher education is a key transitional period, particularly for international students and those living out of home for the first time^(8)^. Students are often faced with academic and financial pressures associated with tuition and accommodation, sometimes compounded by a lack of family support and knowledge gaps in food preparation^(9)^. Evidence indicates FI among students is linked with poorer mental and physical health outcomes including higher BMI and depression^(10–13)^. Studies have identified that student food security barriers are often related to time scarcity, insufficient money for adequate, healthy foods^(14)^ and a lack of access to culturally appropriate foods, particularly for international students^(15)^. This can lead to the sacrifice of nutritional quality for meals that are more accessible, increasing obesogenic behaviours^(10,16)^. Campus food environments are also influential in determining student dietary choices, and there is often a lack of healthy and affordable food options available on higher education campuses^(17,18)^. FI in students has also been associated with poorer academic outcomes including lower grade point averages and class attendance^(10,19,20)^.

Patterns of FI amongst students are often tied to social and economic inequities. Several studies have shown how FI disproportionally affects students because of factors such as race^(21,22)^, international enrolment^(7,23,24)^, gender^(24)^, socio-economic status^(25)^, living away from home^(26)^ and existing household FI^(14)^. Recent research has also demonstrated that food-insecure students may simultaneously experience other insecurities related to basic needs and housing^(12,19)^.

Increased awareness of this issue has prompted higher education institutions to develop programmes that attempt to meet students’ food needs. The most common solutions offered by higher education institutions include campus food pantries, student meal plans and financial assistance programmes^(9)^. Whilst these efforts play an important role in increasing access to food, the persistently high prevalence of FI among students, suggest they may not fully meet the needs of students. Most food security programmes are food relief-centred^(27)^, which can risk perpetuating the stigmatisation of already marginalised food-insecure students^(28)^. There is also evidence that existing food-relief programmes often do not meet nutritional needs^(29)^. This points to the need for more multidimensional strategies including those at an institutional governance level to support and prioritise FI^(9)^. Such strategies may include policies and procedures, that allow for monitoring, investments and infrastructure to support effective and sustainable programmes^(30)^.

Although there is burgeoning research in this area, there tends to be a strong focus on identifying the extent of the problem through passive forms of engagement with students rather than in the development of solutions. Despite being key stakeholders and end users of the FI initiatives, students’ voices, perspectives and lived experiences are often underexplored^(31)^. Effective engagement with a variety of stakeholders, including end-users, in the design and development of solutions may offer more inclusive and sustainable strategies^(32,33)^. This approach may also strengthen student agency, a pillar of food security recently defined by Clapp et al. as ‘the capacity of individuals to contribute meaningfully to the processes that govern their food systems’^(34)^. By engaging with students who are directly affected by FI, through participation in research and decision-making processes, institutions can better tailor initiatives to address specific needs and barriers.

Research methodologies that emphasise participation are becoming increasingly popular in addressing public health issues^(33,35)^. Participatory research is a broad term describing research designs, methodologies and frameworks used to elicit ‘direct collaboration with those affected by the issue being studied for the purpose of action or change’^(35)^. Participatory research methods can vary, from conventional surveys and focus groups to more involved methods such as community-led research^(36)^. Participatory research methods can allow for co-design, where stakeholders collaborate with researchers to design and develop solutions or interventions that meet the needs of the population being studied, ensuring outcomes are relevant, effective and tailored to their specific context.

With the increasing recognition of student FI, higher education institutions are poised to integrate new strategies and institutional policies, that should embody students’ experiences of FI and their perspectives of effective solutions. This scoping review aims to describe and evaluate evidence of student participation in research and identifies strategies related to FI in the higher education setting. The following research questions guided this scoping review: (1) Is there evidence for the integration of co-designing methodologies in research aimed at addressing student FI? (2) What strategies and outcomes have resulted from utilising these methodologies? (3) Are there any barriers to student participation in collaborative research methodologies?

## Methods

A scoping review was selected due to its capacity for broad exploration of the existing literature, particularly given the nascent nature of research on campus FI, as evidenced by the lack of comprehensive reviews. This scoping review follows Arksey and O’Malley’s six-stage methodological framework^(37)^. The review was conducted based on the Preferred Reporting Items for Systematic Reviews and Meta-Analyses extension for Scoping Reviews checklist (see Supplementary File 1)^(38)^. There was no quality appraisal as per scoping review guidelines^(37,38)^. A title and abstract review were conducted by one reviewer (T.S.), and articles that did not meet the defined inclusion criteria were excluded. Full-text review was then undertaken by three reviewers (T.S., N.F., G.G.). In the case of disagreement, reviewers discussed eligibility and came to a collective agreement. A protocol further detailing the study’s methodology and search strategy is publicly available through Open Science Framework, doi: https://doi.org/10.17605/OSF.IO/573NT.

### Search strategy and study selection

A preliminary literature search was conducted in Ovid MEDLINE and Google Scholar to identify key search terms. Medical subject headings (MeSH) terms were selected accordingly. A search strategy was developed and piloted based on key terms identified and concepts derived from the research questions with the assistance of a clinical librarian. A systematic search using three academic databases, Ovid MEDLINE, Scopus and Web of Science was then conducted. An example of the search strategy is presented in Table 1. The scoping review considered all peer-reviewed primary studies and grey literature published from January 2010 to May 2024, with the exclusion of review articles. Studies were selected if they met the criteria in Table 2.

**Table 1.** Search terms and concepts developed for Ovid MEDLINE database

Concept	Keywords
1.University	Universities (MeSH Term)* Students/ (MeSH Term)* OR universit* OR college* OR tertiary OR higher education OR campus* OR student OR undergraduate OR postgraduate
AND	
2.Participation	Social participation (MeSH Term)* Consumer advocacy (MeSH Term)* Stakeholder participation (MeSH Term)* co design* OR co develop* OR participat* OR engage* OR involve* OR decision making community based participatory research OR citizen science OR ‘action research’ (social or stakeholder or community or student) adj3 (participat* or perception* or experience* or engage* or advoca* or decision making or involve*) polic* or guideline* or governance or agenda
AND	
3.Food insecurity solutions	Food supply (MeSH Term)* Food insecur* OR access to healthy foods OR food secur* OR nutrition food adj2 (security or insecurity or access* or availab* or afford* or environment*)

*Includes all subheadings.

**Table 2.** Inclusion and exclusion criteria for academic and grey literature

Inclusion	Exclusion
– Target population: higher education students (of all age ranges and enrolment types) – Setting: higher education institutions including universities and colleges – Outcomes: food security solutions/strategies/initiatives/policies/projects – Intervention: participatory research methods or any evidence of student participation – Published literature – Countries: All – English – Published between 2010–May 2024 – Primary studies, practice briefs and case studies, and policies, reports and strategic plans	– Not related to higher education or tertiary education settings – Non-English – Published before 2010 – Secondary studies i.e. review articles

A grey literature search plan was developed for Google and Google Scholar. The advanced search feature was used to enter search terms and limit the search from January 2010 to May 2024. To further narrow the search, filtering for English language and PDF file types was applied. The search strategy included the following search terms: (university OR higher education OR college OR campus) AND (food security OR food insecurity OR food access OR food environment) AND (engagement OR participatory action OR engagement OR co-design OR co-develop OR participatory research). Due to the complexity of screening all retrieved results from Google and Google Scholar, the relevancy ranking in these search engines brought the most relevant results to the top. Then, the first ten pages of each search hit (∼100 results in total) were screened, including the title and if provided, the summary or abstract. The search strategies including the filters applied, number of hits and search terms were recorded in a Microsoft Excel spreadsheet. After screening, potentially eligible grey literature was uploaded into a Microsoft Excel spreadsheet before undergoing a full-text review. Grey literature was in the form of policies, strategic plans and reports that met the criteria in Table 2 and was from English-speaking countries including but not limited to Australia, New Zealand, Canada, United Kingdom and United States.

### Key terms and definitions

Studies were assessed for their inclusion of key concepts related to the research questions. Such concepts are outlined here along with their definitions and the approach used for assessment.

The United Nations Committee on World Food Security describes food security as ‘the physical, social and economic access to sufficient, safe and nutritious food that meets their dietary needs and food preferences for an active and healthy life’^(5)^.

In this scoping review, studies addressed any aspect of food security associated with the six pillars of food security – availability, access, utilisation, stability, sustainability and agency^(34)^. Recognising that food and nutrition security often overlap, studies involving nutritional interventions were considered in the review process.

Higher education, also known as post-secondary education or tertiary education, refers to any form of education that takes place after the completion of secondary education. The structure of higher education may vary by country; however, it generally includes institutions such as universities and colleges that offer academic, professional or vocational qualifications.

Co-designing can promote meaningful participation evoking individuals’ or groups’ perspectives, insights and ideas, with their input being taken into account in shaping outcomes, actions or policies^(39)^. The level of participation is often proportional to the degree of influence students have in the research and decision-making process^(36)^. The International Association of Public Participation’s (IAP2) Spectrum of Participation was adapted to assess studies and define a specific level of student participation in the research (see Table 3)^(40)^.

**Table 3.** Level of student participation in research for food security solutions, adapted from IAP2 spectrum of public participation^(40)^

Level of participation	Criteria
Consultative	Students contribute their opinions, feedback, thoughts, perspectives or experiences relating to the problem and potential solutions. Includes but not limited to surveys, focus groups and interviews
Involvement	Students are actively involved throughout more than one of the research stages (i.e. through workshops and ongoing meetings)
Collaborative and partnership	Students are involved as partners in the development and identification of solutions. They may be referred to as ‘co-researchers’ or ‘co-designers’ (i.e. participatory action research)
Empowerment and student-led	Students lead research processes are involved in decision-making, designing and implementing solutions

Participatory research sometimes incorporates specific methodological approaches, utilising conceptual frameworks, models or theories such as participatory action research, community-based participatory research, citizen science and participatory evaluation^(36)^.

### Data extraction and analysis

Data extraction variables were selected, and a table in Microsoft Excel was iteratively developed and piloted. Data extracted from the literature included the following information: author, publication year, title, document type, country, study design and methods, study objective(s), setting, participatory model or framework, stakeholder involvement or partnerships, student demographic characteristics, outcome(s), student mode of participation, stage of research cycle, study findings (key strategies), student perceptions and limitations and barriers to student participation. Data were assessed based on the participation level (Table 3) and the step of the research process where participation occurred. The five-step research process was based on the National Health and Medical Research Council’s research cycle^(41)^. Additionally, the themes, tools and features of participatory methods were organised into the domains specified by Duea et al. (2022): (1) Engagement and capacity building; (2) Exploration and visioning; (3) Visual and narrative; (4) Mobilisation and (5) Evaluation^(32)^. A simple content analysis approach was used to categorise the strategies and solutions identified in the included studies^(42)^. Using Microsoft Excel, one author (T.S.) repeatedly read the extracted text and inductively created descriptive codes that described the type of strategy or solution. Categorisation of strategies and solutions was primarily inductive; however, the socio-ecological model was considered during this process, as the resulting categories loosely aligned with its recognition of multiple levels of influence affecting students. Given the interpretive nature of this analysis and the author’s familiarity with the topic of FI, which may have influenced the interpretation of strategies identified, academic researcher reflexivity was considered to account for the author’s familiarity with the topic and academic background. There was regular consultation with co-authors (N.F. and G.G.) who acted as secondary reviewers to this coding and categorisation process. This helped with potential uncertainty and bias around interpretation, supporting the grounding of findings in the data rather than being influenced by one author’s perspective.

## Results

**Figure 1.**
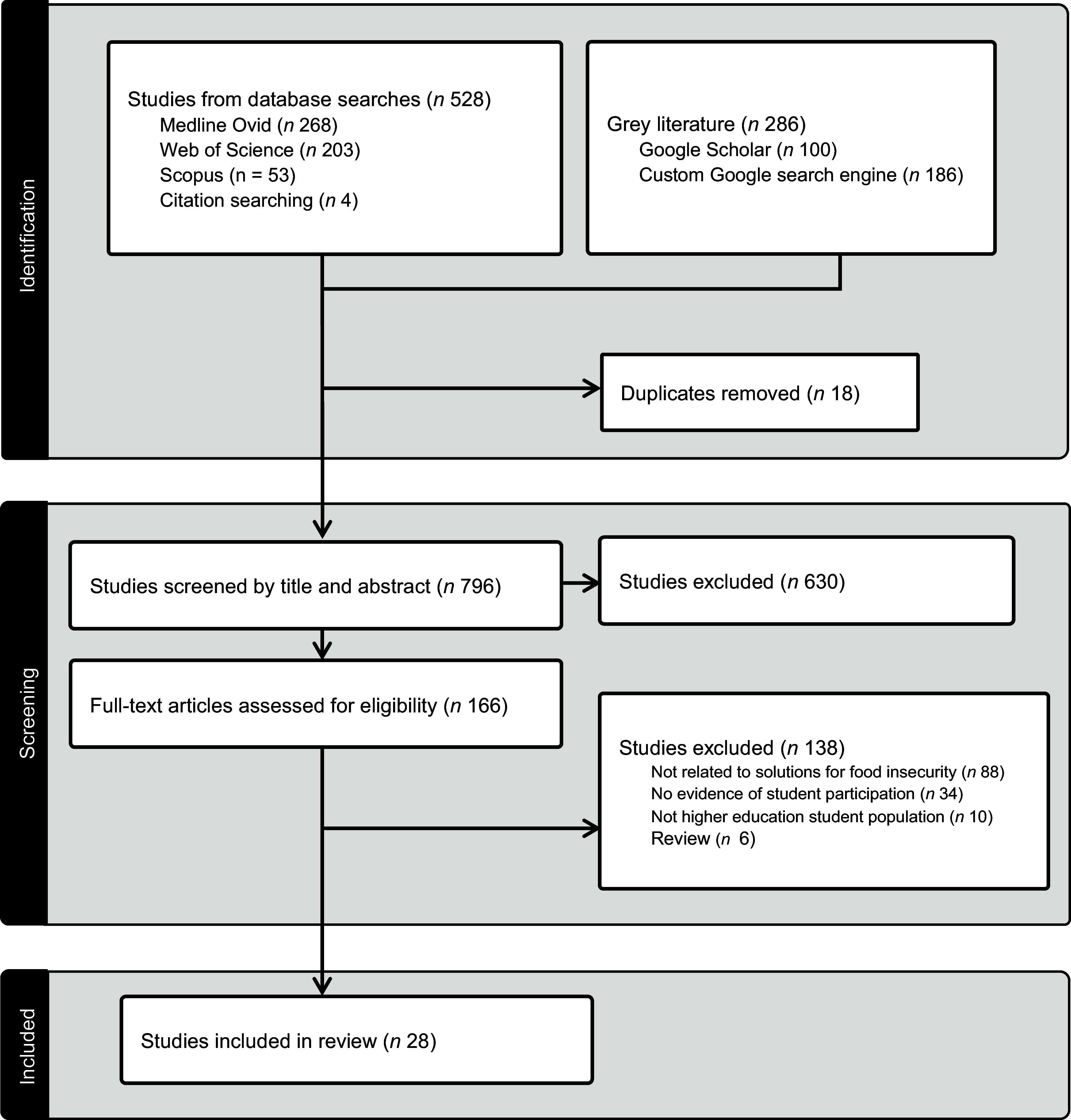
PRISMA flow diagram: co-designing solutions for addressing student food insecurity.

As shown in Figure 1, there were 528 results generated from the primary search strategy in three academic databases and citation searching. A total of 814 articles were screened, including 286 results from grey literature sources. After excluding 648 articles, the remaining 166 underwent full-text review by three reviewers (T.S., N.F. and G.G.). There were 138 articles excluded with reasons documented, resulting in twenty-eight studies included in the review. Findings related to the study characteristics; student participation; stakeholder partnerships and collaborations and outcomes; barriers to student participation and strategies will be provided in this section.

### Study characteristics

The studies were heterogeneous in study design and scope. These characteristics are detailed in Table 4. The majority of studies were conducted in higher education settings in the United States (*n* 22, 79 %), and the remaining in Australia (*n* 4, 14 %) and Canada (*n* 2, 7 %). The publication dates of included studies ranged from 2017 to 2023, with the majority of studies conducted between 2020 and 2024 (93 %). The study designs were classified as either qualitative (*n* 19, 68 %) or mixed methods (*n* 9, 32 %). The included grey literature was in the form of reports, strategic plans and panel summaries where co-design methodologies were utilised. Of the studies included, the majority were situated in the university setting (*n* 26), and the remaining were in a community college (*n* 1) and a Technical and Further Education (TAFE) institute setting (*n* 1). There were eleven studies that specifically screened students for FI who were involved in the identification of solutions in some way (39 %) and five that explicitly involved students who had been identified as food insecure (17 %). The measures used to screen food security status varied across studies. These included the United States Department of Agriculture Six-item Short Form Food Security Survey Module (*n* 4), United States Adult Food Security Survey Module (10 item) (*n* 2), Household Food Insecurity Access Scale (*n* 1), self-identified food insecurity (*n* 2) and one study where the measure was unclear (*n* 1).

**Table 4.** Summary of study characteristics

	Sub-categories of study characteristics	*n*	%
Study design	Qualitative	19	68 %
	Mixed methods	9	32 %
Publication year	2010–2014	0	0 %
	2015–2019	2	7 %
	2020–2024	26	93 %
Country	United States	22	79 %
	Australia	4	14 %
	Canada	2	7 %
Higher education setting	University	26	93 %
	College	1	3·5 %
	Other^*^	1	3·5 %
Number of participants	1–10	3	11 %
	11–50	12	42 %
	51–100	5	18 %
	> 100	3	11 %
	Not reported	5	18 %
Gender	Majority female (> 60 %)	11	39 %
	Majority male (> 60 %)	0	0 %
	Majority LGBTQIA+ (> 60 %)	1	3 %
	Mixed (equal)	4	14 %
	Not reported	13	46 %
Education level	Majority undergraduate (> 60 %)	15	54 %
	Majority postgraduate (> 60 %)	0	0 %
	Mixed (equal)	3	11 %
	Not reported	10	35 %
Enrolment status	Majority domestic (> 60 %)	2	7 %
	Majority international (> 60 %)	3	11 %
	Mixed (equal)	2	7 %
	Other^†^	1	3 %
	Not reported	20	71 %
Screened for food-insecure participants	–	11	39 %
Included exclusively food insecure students (100 %)	–	5	17 %

* Technical and further educational institution.

† Military-connected students.

### Student participation

The type and degree of student participation varied across the studies which is detailed in Table 5. Twenty-three of the twenty-eight studies explicitly mentioned the number of students involved, which ranged from 2 to 339 students. Most studies had between eleven and fifty participants (*n* 12, 42 %).

**Table 5.** Characteristics of included studies for student participation in food security research

Author/ Year	Setting (higher education institution)	Participatory methodology	Model/ theory	Partnerships, collaborations and stakeholders	Collaborative themes and tools (adapted from Duea et al. 2022)	Objective(s)
Qualitative methods						
Ahmad et al. 2020	CA (McMaster University)	Student panel Health forum	Evidence, insight and action	Students and unidentified university stakeholders	Engagement and capacity building Stakeholder involvement Mobilisation Student panel	To convene a student panel on strengthening efforts to support student FS
Anderson et al. 2022	US (University of Tennessee)	Semi-structured interviews	NR	Students and researchers	Exploration and visioning interviews	To explore the meaning of FI and its impact on students’ lived experiences and food decisions, facilitators and barriers to food access and students’ proposed solutions
Brand 2023	US (University of San Francisco)	Peer in-depth interviews Journal entries Testimonials	PAR	Student researchers, university research staff and students	Exploration and visioning Group planning process Mobilisation Action planning Dissemination Evaluation Collaborative evaluation	To collaborate with students to deepen their understanding of student FI, develop innovative strategies and solutions
Conrad et al. 2022	US (Mississippi State University)	Focus groups Open-ended questionnaire	NR	Students and researchers	Exploration and visioning Stakeholder priority setting Exploration and visioning Focus groups	To identify students’ perceptions about food access resources and explore students’ expressed needs from the university
DePorter et al. 2023	US (University of Wisconsin-Madison)	Case study Student-led initiative	NR	Students, organisation partners with local grocery stores and research farms	Engagement and capacity building Stakeholder involvement	To describe the student-led creation and management of the organisation, Campus Food Shed, and its daily operations and challenges and opportunities for growth
Dhillion et al. 2019	US (University of California)	Focus groups	NR	Students and researchers	Exploration and visioning Facilitated focus group discussions	To examine student perceptions of the campus food environment, existing solutions and potential future solutions
El Zein et al. 2022	US (University of Florida)	Semi-structured and in-depth interviews	NR	Students and researchers	Exploration and visioning Interviews Evaluation Participatory evaluation	To explore students’ perceived barriers to using an on-campus food pantry and identify student-suggested solutions
Henry et al. 2023	US (University of North Texas)	Applied ethnographic approach In-depth and semi-structured interviews	NR	Student researchers, students and researchers	Exploration and visioning Facilitated focus group discussions	To explore the perceptions and experiences of FI students who identify as LGBTQIA+ and identify inclusive solutions
Jeffrey et al. 2021	AU (University of Melbourne)	Interviews Student-led research	NR	Student co-researchers, students and academic researchers	Engagement and capacity building Stakeholder involvement	To carry out a research project with student co-researchers to interview students experiencing FI. The results were used in a podcast
Kim et al. 2022	CA (University of British Columbia)	Facilitated dialogues Booths and pop-up installations Online survey Student-led projects On-going consultations and meetings Panel	Community-based PAR	Students, faculty, staff, student group leaders, student community developers and researchers, administration, president, sustainability coordinators, and health promotion specialist	Engagement and capacity building Working advisory group Stakeholder involvement Exploration and visioning Facilitated focus group discussions Audio diaries Life mapping Interviews Evaluation Participatory evaluation of pilot	To identify current gaps in FS support for students and produce reflections and recommendations, all of which will inform the future development of a Community Food Hub on campus
‘Healthy’ et al. 2023	AU (University of Tasmania)	Sustainability surveys Environmental audit	PAR	Students, multiple faculties, staff, researchers, sustainability committee, community members, student association, gardening and cooking societies and Health, Nutrition and Sustainability Working Group, Tasmanian Food Security Coalition	Engagement and capacity building Stakeholder involvement Partnership with an FS coalition Exploration and visioning Group planning process	To transform the campus food environment to one that is healthy, sustainable and equitable through an established strategic plan and a rights-based approach
OoNorasak et al. 2022	US (University of Kentucky)	Student-led programme Evaluation survey	Socio-ecological model	Students, faculty, researchers, Campus Kitchen students and volunteers	Engagement and capacity building Collaborative programme planning and stakeholder involvement Evaluation Participatory evaluation	1. To evaluate the student-led Campus Kitchen programme 2. To describe behavioural perceptions of students who utilised the Campus Kitchen’s Farm to Fork free meal programme
Porter et al. 2023	US (University of Wyoming)	Student-led project Survey Task force meetings	NR	Co-mentored students, researchers, student organisations and groups	Engagement and capacity building Stakeholder involvement Mobilisation Working task force Meetings	To develop a working task force with multiple diverse student groups to identify priorities, assets and ideas to improve student FS
Rousseau 2023	US (California State University)	Bi-weekly team meetings Praxis	PAR	Students co-researchers, researchers, faculty, programme administrators and university administration	Engagement and capacity building CBPR, collaborative planning with stakeholders Relationship building period Working group Visual and narrative Logic model planning ‘Flexible diary’ data collection	To gain an in-depth understanding of the challenges faced by food and housing-insecure students and empower their strengths and perspectives within the campus community and ensure their voices are reflected in institutional programmes
Shisler et al. 2023	US (North Carolina State University)	Workshops Asset-mapping	Community-based participatory research	Researchers, student organisations, faculty and staff	Engagement and capacity building Collaborative planning with stakeholders Steering committee Visual and narrative Asset-mapping Mobilisation Mobilisation of campus community	To mobilise the campus community and identify solutions to address the root causes of FI and other basic needs insecurity among students
Schinkel et al. 2022	US (University of Wyoming)	Survey Focus groups Co-designed research processes	NR	Military-connected students, Food security task force (run by staff, students, faculty and administrators), researchers	Engagement and capacity building Stakeholder involvement Exploration and visioning Facilitated focus group discussions Mobilisation Working task force	To increase knowledge of military-connected student FI experiences and explore potential strategies to address food access
Sampson et al. 2020	US (Universities in Michigan)	Survey Workshop Summit	Feminist action research	Students, staff, programme volunteers and practitioners	Engagement and capacity building Summit Collaborative planning Stakeholder involvement - Community of practice Exploration and visioning Concept mapping Group level assessment Evaluation Participatory evaluation	To address FI among college students through the planning and implementation of the Michigan College Campuses Food Pantry Summit using principles from the feminist action research cycle
Watson et al. 2017	US (University of California)	Focus groups	NR	Students and researchers	Exploration and visioning Facilitated focus group discussions	1. To understand how students perceive, experience and cope with FI 2. To explore opportunities to improve food literacy among college students
Ullevig et al. 2021	US (University of Texas at San Antonio)	Student-led intervention	NR	Students, staff, faculty and researchers	Engagement and capacity building Stakeholder involvement Steering committee	To evaluate and explore students’ experiences implementing FI programmes
Mixed methods						
Abu et al. 2022	US (University in West Texas)	Online survey Focus groups	NR	Students and researchers	Exploration and visioning Facilitated focus group discussions Stakeholder priority setting	To investigate students’ perspectives on the causes of FI, coping strategies and solutions
Adamovic et al. 2022	US (multiple universities)	Survey	NR	Students and researchers	N/A	To identify solutions to campus FI that might be most attractive to, and appropriate for, food-insecure students
Ahmed et al. 2021	US (Kingsborough Community College)	Student-led intervention Survey	NR	Students, student leaders, staff and researchers	Engagement and capacity building Stakeholder involvement Evaluation Participatory evaluation	To increase students’ awareness and understanding of FI through a student co-designed ‘Playing with Our Food’ intervention
Barr et al. 2022	US (two land-grant universities)	Student-led intervention Stakeholder interviews Needs assessment	Community-based participatory research	Student researchers, Student Wellness, Food Pantry Manager, Registered Dietitian, College Dean and course instructors	Engagement and capacity building Community advisory board Exploration and visioning Nominal Group Technique Needs assessment and prioritisation	To utilise a CBPR college course with student research partnerships to collect needs assessment data from students and serve as a community advisory board to assist in the development of an intervention
Fortin et al. 2021	US (University of Kansas)	Focus groups Interviews Survey	NR	Students and researchers	Exploration and visioning Facilitated focus group discussions Interviews	To gain student perspectives on the complexity of FI by exploring eating patterns, food assistance and health
Frank 2022	US (University in Philadelphia)	Focus groups Survey Stakeholder meetings	Mobilise, assess, plan, implement and track	Stakeholder meetings: students, staff, faculty and researchers, campus food service provider Collaborations: Campus dining services, student life and leadership, associated students incorporation, and the information technology division	Engagement and capacity building Stakeholder involvement Exploration and visioning Facilitated focus group discussions Needs assessment Evaluation Feedback sessions with stakeholders	To develop, implement and evaluate an electronic learning management system to address student FI
Soederberg Miller et al. 2023	US (California public university)	Focus groups	NR	Researchers and students	Exploration and visioning Facilitated focus group discussions	To examine at-risk of FI students’ perceptions and meal preparation attitudes and desired future practices for how the college environment could support student efforts to prepare meals at home
Supski et al. 2023	AU (William Angliss Institute)	Seminar Survey Student subject engagement Student-led programmes	NR	Student researchers, students undertaking a subject Seminar Two other university stakeholders	Engagement and capacity building Stakeholder involvement Seminar	To identify the extent of FI among students and to identify and implement strategies and solutions
Waite et al. 2023	AU (Monash University)	Survey In-depth semi-structured interviews	NR	Students and researchers	Engagement and capacity building Stakeholder involvement	To provide an evidence base for the development of socially and culturally appropriate solutions to FI experienced by international university students

	Level of student participation				Student participation in the research cycle					
Author/ Year	Consultative	Involvement	Collaborative	Empowerment/student-led	1. Identification of solutions	2. Design & develop of research/ solutions	3. Conduct/implement research/ solutions	4. Dissemination of results	5. Evaluation/reflection of solutions	Outcome(s)
Qualitative methods										
Ahmad et al. 2020		☒			☒					Students’ involvement in the identification of solutions to FI to inform future decision-making
Anderson et al. 2022	☒				☒					Recommendations for the design of comprehensive policy, educational and nutritional interventions
Brand 2023			☒		☒	☒	☒	☒	☒	1. Student awareness and understanding of FI 2. Identification of student-suggested strategies to address FI 3. Student leadership, action and advocacy for FI justice 4. Evaluation of action plans with relevant stakeholders
Conrad et al. 2022	☒				☒				☒	1. Inform the development of resources and interventions that are in line with students’ needs, enhancing resource utilisation to increase FS status 2. The evaluation of existing resources for campus FI (meal plans)
DePorter et al. 2023				☒			☒			1. To spread awareness of student FI 2. To inspire other higher educational campuses and community groups to establish similar sustainable food distribution systems run by students
Dhillion et al. 2019	☒				☒				☒	1. Recommendations for solutions to FI suggested by students. 2. The critique of existing FI solutions (meal plans)
El Zein et al. 2022	☒				☒				☒	The identification of student-perceived barriers and solutions to the existing campus food pantry which allowed for programme improvements
Henry et al. 2023	☒		☒		☒					Recommendations for inclusive university-level solutions for LGBTQIA+ students experiencing FI
Jeffrey et al. 2021				☒	☒	☒	☒	☒		1. Students’ results were used in a podcast to increase awareness and improve advocacy for student FI 2. Recommendations for strategies that address the availability of affordable and healthy food on campus
Kim et al. 2022			☒	☒	☒	☒	☒		☒	1. The implementation of the initiative ‘Food Hub’ as a sustainable campus food system 2. The development of a partner network between student services to coordinate holistic support system for students within the Food Hub 3. Increased community awareness around food access, FS and the Food Hub
‘Healthy’ et al. 2023			☒		☒	☒	☒			1. Revised guidelines, policies and procedures directing university activity and decision-making directly towards improved FS outcomes 2. An established memorandum of understanding between the university and the existing Tasmanian Food Security Coalition 3. Raised awareness among university community 4. Created a shared vision among all new and existing initiatives and activities associated with the food system 5. Bi-annual campus food environment audit
OoNorasak et al. 2022	☒			☒					☒	Recommendations for programmes similar to Campus Kitchen, that aim to reduce food waste and FI in communities and college campuses
Porter et al. 2023				☒	☒	☒	☒	☒		1. A sustainable, equity working group with a central focus on FI 2. A guide for creating a food share cabinet 3. Student awareness and understanding of FI and resources 4. Student leadership, action and advocacy for addressing inequities and FI
Rousseau 2023			☒		☒	☒	☒	☒		1. Build partnership between stakeholders (students, faculty and programme administrators) 2. Recommendations through priority action areas 3. Stakeholder identification and strategies for advocating to engage campus decision-makers
Shisler et al. 2023				☒	☒	☒				1. Mobilisation of campus community for FI action and advocacy 2. Collaborative identification of multi-level solutions to FI 3. Determine priorities for action and policy to address students’ needs 4. Articulate the needs and assets of various campus stakeholders in addressing FI
Schinkel et al. 2022	☒		☒		☒	☒				Recommendations for institutional actions to improve FI and insight into food access resources for military-connected students
Sampson et al. 2020			☒		☒	☒			☒	1. Co-designed workshop for campus food pantry evaluation 2. Formation of a regional community of practice 3. A panel of policymakers and community leaders to discuss potential programmatic and policy actions
Watson et al. 2017	☒				☒					Recommendations for student-suggested solutions for improving FI on campus and food literacy skills
Ullevig et al. 2021		☒			☒	☒	☒		☒	Recommendations and ‘lessons learnt’ for other higher educational institutions that may be considering implementing campus garden and food pantry to address student FI
Mixed methods	Mixed methods									
Abu et al. 2022	☒				☒					Recommendations for multi-level solutions to FI on campus suggested by students
Adamovic et al. 2022	☒				☒					Recommendations for future university-level solutions including policy and programme improvements that are acceptable and appropriate for food-insecure students
Ahmed et al. 2021				☒			☒		☒	1. Student awareness and understanding of FI 2. Student leadership, action & advocacy for FI justice 3. Recommendations for student-suggested strategies and solutions for greater FS on campus
Barr et al. 2022				☒	☒	☒	☒	☒	☒	A peer-led intervention designed to improve student diet quality, FI and student awareness of existing resources
Fortin et al. 2021	☒				☒					Recommendations for future programmes with an intended audience of university administrators and public health practitioners
Frank 2022		☒			☒	☒	☒		☒	1. The programme ‘Free Food on Campus!’ initiative was co-designed, implemented and evaluated with stakeholders to alleviate FI and improve food waste 2. Improve food culture at the university
Soederberg Miller et al. 2023	☒				☒					Recommendations derived from student-suggested solutions for campus-provided support for meal preparation to relieve FI
Supski et al. 2023			☒	☒	☒	☒	☒	☒	☒	1. Increase institution and public awareness of FI 2. Evaluate student perceptions on campus food environment and existing FS solutions 3. Recommendations for future strategies and solutions targeting campus FS 4. Development of designated staff (project officer) to provide administrative and operational support for campus food programmes
Waite et al. 2023	☒				☒					1. Recommendations for culturally relevant and responsive system-level changes including policy and interventions at the university to address FI among international students 2. To raise awareness of the challenges around FS experienced by students

CA, Canada; FI, food insecurity; FS, food security; NR, not reported; PAR: participatory action research; LGBTQIA+, Lesbian, gay, bisexual, queer, intersex; AU, Australia.

The objectives of all studies were similar in that the purpose of student participation was to identify and utilise students’ experiences, perspectives and ideas for solutions. An additional reason for student involvement was that the study involved student-led interventions and programmes (*n* 4). In twelve of the twenty-eight studies, students participated in the research process for the purpose of evaluating or reflecting upon existing solutions to student FI (*n* 12).

There were three distinct student subpopulations explicitly involved in three studies: international students^(43)^, military-connected students^(44)^ and students identifying as LGBTQIA+^(45)^. For demographic characteristics, 39 % of the studies had a majority female (*n* 11) and 54 % undergraduate students (*n* 15) reported. Many studies provided evidence of student participation; however, there was still a significant amount of unreported data on gender (46 %), education level (35 %) and enrolment status (71 %).

Ten studies specified a participatory model or framework in the study design. This included participatory action research (*n* 3), community-based participatory research (*n* 3), feminist action research (*n* 1), socio-ecological model (sem) (*n* 1), ‘Mobilize, Assess, Plan, Implement and Track’ (MAP-IT) framework (*n* 1) and an ‘Evidence, Insight, Action’ framework (*n* 1).

For the level of participation, seventeen out of twenty-eight studies had involved students in more than one step of the research cycle. However, only three studies reported student participation across all five steps, indicating continuous engagement. Evidence of co-design and co-development was found in thirteen of the twenty-eight studies. There were six studies that had evidence of student participation from the first step: identification of a solution, to the fourth step: the dissemination of results.

Regarding the four participation levels, the most common was the minimum, consultative, identified in thirteen of twenty-eight studies. The highest level of participation, student empowerment, was identified in nine of twenty-eight studies, with three of these studies specifically noting student-led research.

Three of the twenty-eight studies had the second lowest level of participation which was involvement, and eight of twenty-eight studies had the second highest level of participation which was collaborative. There were five studies that had more than one level of participation recorded. This was because the study was led by student researchers who had conducted research processes on other students, for instance, student-led interviews or focus groups. For conventional participatory research methods, studies included utilised surveys (*n* 13), focus groups (*n* 8) and individual interviews (*n* 6) to collect student’s experiences, opinions and ideas towards solutions.

### Stakeholders, partnerships and collaborations

The types of partnerships, collaborations and stakeholders also varied across studies. Ten of the twenty-eight studies included only students and researchers in the research cycle. Whereas most of the studies had evidence of collaboration and partnership among multiple stakeholders (*n* 18, 64 %). These stakeholders included student co-researchers (*n* 7), student organisations and groups (*n* 3), university staff including administrators and faculty (*n* 8), sustainability coordinators (*n* 2) and food programmes staff and volunteers (*n* 3). Three studies also showed evidence of engagement with clinical and public health staff including practitioners^(46)^, health promotion specialists^(47)^ and registered dietitians^(48)^. As shown in Table 3, some studies had evidence of engagement with stakeholders through specific organisational structures or groups such task forces^(44,49)^, coalitions^(50,51)^, group seminars^(52)^, panels^(47,53,54)^, working advisory groups^(47,48,55)^ and steering committees^(56,57)^.

### Outcomes

The most common outcomes were recommendations for strategies including policy and programme development (*n* 23), evaluation of existing programmes (*n* 5), increased awareness of student FI among the university community (*n* 11) and enhancing advocacy efforts (*n* 5). For studies with higher levels of student participation, particularly, collaboration, empowerment or student-led, there was often more than one outcome reported. Such outcomes included increased student awareness and understanding of FI^(43,47–52,54,58,59)^, student leadership, action and advocacy for food justice^(49,51,54)^, the development of campaigns^(50,52,54)^, working groups, partner networks and coalitions^(44,47,49,50)^.

### Barriers to student participation

Understanding the barriers to student participation is important for improving participatory methodologies for future research related to food security. Barriers to student participation were identified in nineteen of the twenty-eight studies. One of the barriers was related to social desirability bias linked to the stigma of being identified as food insecure, which affected participation in the research^(60)^. Conformity bias was also noted particularly in focus group settings, where students may have refrained from sharing unpopular individual experiences, resulting in potentially homogenised suggestions on solutions^(61,62)^. Both types of bias hindered open communication, leading to lower participation rates, less meaningful participation and results that were potentially not representative of the actual experiences and needs of food-insecure students^(60–62)^. Co-researcher students in one study also felt uncomfortable seeking out food-insecure students for interviews due to fear of further stigmatising their food-insecure peers^(51)^. Although 43 % of studies (*n* 12) screened students for FI, one study avoided this process to specifically reduce the risk of stigmatising student participants^(56)^.

Student-led research studies (*n* 3) noted that barriers to sustained engagement and project task completion were most likely due to a lack of student availability, busy schedules, high student turnover rates and long-term communication issues^(47,48,57)^. There were also challenges to engaging in stakeholder collaboration between and within multiple campus stakeholders and student groups to be involved in implementing food security initiatives^(53)^.

Four studies reported limitations of poor representation of diverse student groups including minority groups whose experiences of food security may be compounded by other experiences of stigma and discrimination^(31,45,47,48,56)^. In Kim et al.’s study (2022), the project outreach efforts failed to reach diverse student groups due to capacity and time constraints^(47)^. Additionally, the limited representation of student groups in the needs assessment further affected the final determination of a campus-wide programme in Barr’s study (2023)^(48)^.

### Strategies and solutions

Seven key categories emerged for strategies and solutions for addressing FI. These include: (1) policy and institutional support; (2) community engagement, strategic partnerships and coalitions; (3) advocacy and awareness; (4) activities and initiatives for engagement with students; (5) student skill and knowledge development; (6) programmes and (7) campus food environment improvements. These are displayed in detail in Table 6.

**Table 6.** Co-designed strategies for higher education institutions to address student food insecurity

Category	Strategies and recommendations
Policy and institutional support	Training and educating staff including academic, faculty, student well-being and support service staff to support students experiencing FI^(45,52,55,56,87)^ Having a centralised support person or team for FI support for students (i.e. Food System Officer or Food Security Officer)^(52,55,56,87,88)^ FS education and research embedded in co-curricular, coursework or dedicated research projects for students^(49,51,52)^, with PAR methodologies to train students as co-researchers^(48,51,55)^ Regular monitoring of prevalence, impacts and demographic information to learn inequities and sub-populations experiencing FI^(58,59)^ Providing funding and support for student clubs, societies and organisations for food-specific areas such as nutrition, sustainability, gardening and cooking^(50,55,57)^ Supporting food affordability on campus including healthy options such as fruit and vegetables^(89)^ Prioritising equity at the centre of every FS strategy and policy^(49,50,56)^ Including more systems-based approaches rather than immediate food relief or charity-based models (i.e. institution-run programmes and policies)^(44,50,56)^ Incorporating an FS strategy within broader health and wellbeing policies^(50,59)^ Connecting with local urban agriculture groups to advise sustainable procurement policies^(50,52)^ Healthy food policy for on-campus food outlets, vendors and vending machines^(50,52)^
Community engagement, strategic partnerships and coalitions	Convening a student panel to discuss FI implications and potential solutions^(53)^ Creating a shared vision around respect and dignity for sharing ethos around amplifying students’ voices and lived experiences related to FI^(49)^ Connecting with diverse student organisations representing minority groups such as international, migrant, Indigenous and LGBTQIA+^(49,57)^ Developing community advisory boards and steering committees with student representatives^(48,55,57)^ Prioritising transparency and accountability to support the integration of student feedback into existing policies, programmes and services through open communication channels and student leadership^(47,53)^ Forming campus clubs such as the Food Sovereignty Coalition, with a centralised location for students, faculty, staff and administrators to collaborate^(51)^ Creating collaborative student-led campaigns such as Just Food Collective^(52)^ and working FS task forces partnered with several student organisations^(49)^ Building community–academic partnerships and coalitions with academic, and external non-profits such as campus food outlets, gardens, dining halls, grocery stores, restaurants and farmers’ markets^(43,50,52,58)^ Using collaborative research methodologies such as PAR to support a non-hierarchical relationship with researchers exploring FS in their institutions^(48,51,55,56)^ Conducting asset-mapping workshops with stakeholders to identify differences in perceived assets and needs^(56)^ Using the principles of ‘Feminist Action Research’ to co-design a campus food pantry summit with local higher education institutions to share ideas, experiences and lessons learnt to improve upon existing programmes^(46)^ Forming a community of practice for the exchange of best institutional practices among higher education institutions and to advocate for local or federal policy reforms^(46)^
Advocacy and awareness	Developing an FS awareness campaign with various actors involved in campus support services^(51,53)^, particularly during orientation weeks^(52)^ Endorsing outreach officers for campus food programmes to visit classes and student groups^(54)^ Using inclusive marketing messages such as testimonials to demonstrate diversity and emphasise the right of FS to prioritise health and not as a charitable resource^(45,51,53,60,62)^ Initiating text-message campaigns, social media pages, and podcasts to normalise discussions around FI and information about resources and support^(45,48,53,58,59)^ Student involvement in advertising of programmes i.e. creative arts and media students^(50,57)^ Student research project findings are presented by students to stakeholders including the university provost and student affairs^(51,55)^ Using PAR methodology to support student agency and empower them to be involved in solutions to social justice issues like FI^(50,51)^ Promoting concerns about the environmental impact of food waste rather than FI with the intent of alleviating the stigma associated with accessing food relief programmes^(90)^
Activities and initiatives for engagement with students	Serving student-led community meals (with structured discussions about FI), booths and pop-up installations^(47)^ Utilising creative engagement methods for visualisation such as logic model planning, flexible diary entries, audio diaries and concept mapping^(46,47,55)^ Convening an intervention within a local ‘Food Day Conference’ with student-led educational games and proactive problem-solving^(54)^
Student skill and knowledge development	Targeting food literacy and nutrition education development workshops run by university nutrition and dietetics students with support from other faculties such as sustainability, food sciences and environment i.e. ‘peer-to-peer’ nutrition workshops^(47,50,89,91,92)^ Implementing a practical one-unit undergraduate life skills course for health-promoting behaviours^(61)^ Designing information and resources for newly arrived international students about where and how to access the ingredients and foods they want to purchase^(43)^ Promoting community cooking classes, recipe information and online meal preparation activities on campus^(31,48,50,87,92,93)^ Providing incentives for students’ kitchen equipment and weekly grocery vouchers^(43,92,93)^ Providing financial management education interventions for healthy eating on a budget^(31,44,62,87,88,93)^ Enhancing garden-based and horticultural knowledge through hands-on workshops^(57)^ Supporting student-led and staffed programmes such as food pantry and gardening volunteers^(46,50,57,91)^ Implementing a ‘work for meal programmes’ for food hub student volunteers to redeem meals^(44)^ Providing online resources for support and information on food relief programmes such as interactive maps that are easily accessible through a QR code, university website or mobile app^(31,49,51,53)^
Programme development	Financial assistance programmes Creating customisable payment plans for tuition and accommodation fees^(53)^ Forming university support groups or mentorship programmes for different issues such as financial strain and FI^(55)^ Alleviating strict eligibility criteria for campus food and financial aid programmes^(53,56,87)^ Food relief programmes Establishing student and community-led food hubs^(47,52)^ Arranging paid, volunteer and course credit student positions^(47)^ Using a spoke-hub model for food hub programmes^(47)^ Partnering with food recovery networks and good food recovery programmes with anonymous registration^(44,90)^
Campus food environment improvements	Establishing an audit and evaluation strategy for the campus food environment^(50,53,58)^ Increasing access to kitchen spaces on campus for food preparation resources such as microwaves^(45,50,52,55,61)^ Implementing on-campus grocery providing subsidised healthy food options^(44)^ Increasing nutritional information^(89)^ Increasing culturally appropriate food options such as Halal, vegetarian and vegan options^(43,50,61)^ Working with dietitians and cultural experts to devise healthy menus for university-funded food outlets^(52)^ Supporting urban agriculture approach utilising the principles of an edible campus model with campus farms and gardens^(50,57,91)^

FI, food security; FS, food security, PAR, participatory action research; LGBTQIA+, Lesbian, gay, bisexual, queer, intersex.

## Discussion

This was the first review to explore evidence of co-design approaches in research addressing FI in the higher education setting. Overall, the findings suggest that methodologies incorporating these approaches can promote more meaningful participation with students who may be affected by FI which can lead to more unique and inclusive solutions.

This review explored the field of FI research in higher education settings, noting that 95 % of articles were published in the last 4 years. This surge of recent publications indicates a growing recognition of the issue and increased research focus on understanding and addressing FI among students. All included studies were from higher-income countries, predominantly conducted in the United States, where the prominence of campus food security research may be linked with the country’s mainstream food movements supporting food justice and sovereignty^(63)^. Food justice movements emphasise the need for more just food systems, where healthy, sustainable and equitable food is recognised as a human right, while also addressing the root causes and structural inequities that contribute to FI^(64)^. Some advancements in food security research and policy in the United States have led to developments in organisations such as Swipe Out Hunger and the federal food access programme known as the Supplemental Nutrition Assistance Program^(65,66)^.

The degree of student participation in research and decision-making processes varied across studies included in the review. Results found students were mostly engaged in the formative stages of the research cycle involving the identification of potential solutions and need assessments. There was also variation in the level of student participation across the studies. The most commonly reported level of student participation was consultative, which was the lowest level of participation. Consultative participation often involves traditional qualitative research methods such as focus groups, interviews and surveys where students participate in the research to provide their opinions, experiences and feedback on potential strategies and solutions^(40)^. Conversely, 32 % of studies had students engaged in the highest level whereas 11 % of these were student-led.

Nevertheless, evidence of co-design and co-development was present in 46 % of the studies where 11 % had evidence of student participation in all five steps of the research cycle. Continuous engagement with stakeholders throughout each research cycle phase can characterise co-designing and co-producing processes, constituting co-creation^(67)^. Co-creation which involves active engagement at all stages of the research cycle, promotes continued dialogue, power-sharing and reciprocity between stakeholders^(67)^. Although, as revealed in this review, there are some challenges associated with achieving a high level of collaboration. Continuous engagement with students can be difficult to achieve because of a lack of student availability due to busy schedules, high student turnover rates and long-term communication issues. Some collaborative and student-led studies were found to utilise alternative visual and narrative methods for data collection, analysis and interpretation including journal entries, concept mapping and digital storytelling. These methods can adopt strong collaboration with students by aligning with their interests and empowering them to actively engage in the research process^(32,68)^.

The utilisation of participatory research frameworks can guide researchers in co-creating interventions and strategies^(69)^. There were ten studies in this review (36 %) that utilised some form of participatory framework. These frameworks were often incorporated into course curriculums and student research projects where students were trained to use them to perform research on their food-insecure peers. These methodologies can provide the basis for effective engagement but can also facilitate community capacity building^(32)^. Participatory research frameworks can facilitate individual’s understanding of the problem and its manifestations in their communities, whilst also providing opportunities for skill and knowledge development to create new solutions^(70)^. Community engagement and agency are essential for food justice, decision-making and transformative food system changes^(64)^. This involvement in research processes can also have lasting impacts on community members including empowerment^(71)^. Brand (2023) highlights how participatory action research motivated students to pursue food justice beyond the scope of the study, creating a sustained interest in addressing social justice issues^(51)^. Similar outcomes were observed in a study utilising the community-based participatory research methodology, where student involvement in the Food Dignity Project led to improved values and attitudes towards the food system and enhanced students’ ability to effect change in the local community^(72)^.

Developing strong partnerships was found to be critical for long-term collaboration and the development of sustainable solutions^(47,48,55)^. Rousseau’s study^(55)^ included a 4-month relationship-building period where students could collectively express their experiences in confidence during regular meetings, leading to a sense of mutual support and empowerment. Unlike traditional research methods where participation is often one-off, effective co-design can prioritise relationship and trust-building, particularly when working with marginalised communities and addressing sensitive topics^(73,74)^. Partnerships with students and university stakeholders were often solidified through working groups, task forces and committees which could provide an ongoing communication channel and representation of key stakeholders. Creating these partnerships requires the identification of relevant stakeholders, creating a shared vision and developing a set of values and goals^(75)^. Findings from this study show that this process can be facilitated through exploration and vision strategies such as group planning processes, stakeholder priority setting, asset mapping and comprehensive needs assessments^(32)^. At the University of California, Fanshel and Iles (2021) utilised foodscape mapping with student collaborators, a strategy which led to the development of advocacy projects and changes in the campus food system policy^(76)^. Furthermore, collaborations across academic faculties and disciplines including nutrition and dietetics, public health, agricultural, environmental and social sciences are key to ensuring a comprehensive approach to supporting health and well-being, sustainability and the campus food environment. One key multidisciplinary strategy involved engaging media and creative arts students in promoting and advertising FS programmes, resources and information developed by students and staff from health and nutrition-related facilities^(50,57)^.

Successful coalitions form relationships between internal (i.e. relationships between students and researchers) and external entities^(77)^. The review found evidence of partnerships with local expertise such as established food security coalitions, agricultural groups, local community members, non-profit organisations and other universities to be beneficial in sharing knowledge and increasing capacity for solutions. Community-university partnerships have proven valuable in driving social change within food systems, building community skills and forming a community of practices^(78)^.

While collaborative efforts were found to be important, the power to enable systemic changes lies with institutional leaders, who can allocate resources and infrastructure and enforce policies and procedures that shape and support student food security^(30)^. Several strategies were identified at a policy and institutional support level. Some of these include regular monitoring of FI risk among students, educating institution staff and incorporating comprehensive FS policies into existing health and well-being and food service procurement policies. Some of the studies found student suggestions around having trained and dedicated university staff members who could lead initiatives, drive policy changes for the campus food environment and support students experiencing FI. Findings from this review also suggest that student-led research may have a greater impact on institutional governance by enhancing the capacity of student co-researchers to engage in advocacy processes. Students involved in disseminating results through presentations, workshops and meetings with university stakeholders could help leverage the social justice issue to the forefront of the institution’s agenda.

Recent qualitative work has shown how students are often unaware of their own food security status and the resources and support available at their institution^(79,80)^. Consequently, they may lack an understanding of how FI manifests and the wide-ranging implications. This lack of awareness may explain why studies at the consultative level of student participation with limited representation of food-insecure students, generally identified existing, surface-level solutions to address FI. Examples of these solutions found in some of these studies include implementing campus food pantries and distributing grocery vouchers and emergency food aid. Despite the importance of these solutions, they are typically short-term, addressing immediate needs rather than the root causes of FI^(81)^. This highlights the strength of collaborative research methods which can provide opportunities for mutual learning and encourage participants to think critically about the problem, how it affects individuals and how to facilitate effective, systemic changes^(51)^. An additional benefit is the increased awareness and normalisation of FI in the university community, which has also been outlined as a key recommendation for creating more supportive campus environments^(30)^.

One of the barriers to student participation was ensuring a diverse representation. This is particularly challenging given the complexity of food security, which is often intertwined with various other inequities among students. There was a high representation of undergraduate students involved (54 % of the studies), aligning with recent evidence which indicates undergraduate students were at least three and a half times more likely to be food insecure than postgraduate students^(1)^. There was a focus on some vulnerable groups with increased risk of FI in this review such as LGBTQIA+, military-connected students and international students. Empirical research has also identified other at-risk groups including housing-insecure^(19)^, disabled^(82)^, parents^(83)^ and first-generation students^(84)^. Participatory research methodologies with marginalised communities can promote relevant, appropriate and inclusive strategies and solutions^(85)^. Therefore, future student FI research should adopt similar collaborative methodologies to address the needs of other student groups at risk of FI.

Participatory research methods offer an approach to amplifying the voices of vulnerable and marginalised groups in higher education settings, particularly for FI^(86)^. These methods can create supportive environments for developing non-hierarchical relationships and effective collaboration between researchers and participants^(86)^. Studies in this review with higher levels of student participation, for instance, student-led research, often reported less demographic and FI screening. This likely stems from the stigma associated with FI in higher education environments, where explicit screening for FI and demographic characteristics may inadvertently cause students to feel inferior or further stigmatised, especially in group settings like focus group discussions and workshops. To address this challenge while maintaining diverse representation, one effective strategy that was identified was to integrate participatory research projects, campaigns and task forces with existing student organisations and societies that represent diverse student populations. This approach allows for engagement with diverse groups, potentially reducing stigma whilst capturing a range of experiences and perspectives on campus FI.

This scoping review is not without limitations. Firstly, although this review aimed to explore literature from all countries, findings were exclusively from higher-income nations, predominantly the United States, which limits the generalisability of the findings to other regions particularly, lower- and middle-income countries. Although most studies focused on universities, the broad inclusion for any type of higher education institution, such as community colleges, may also limit the generalisability of the findings to unique institutions which often differ between countries. Given the early nature of this research area, the review sought to capture all potential literature by including three academic databases and two grey literature sources. However, since it involves the higher educational setting, the review is unable to capture any internal institutional efforts that are not published or available online. A potential strategy to address this limitation would be to consult with relevant university stakeholders, though this was not feasible within the scope and capacity of this review. We also acknowledge that a more directed content analysis could have been applied to support deductive coding and improve consistency, thereby reducing potential bias in the categorisation process. Future research could consider adopting a more systematic coding framework when exploring solutions to FI in higher education. Furthermore, scoping reviews have innate limitations associated with having a broader scope, meaning that included studies had some degree of heterogeneity in terms of study design and methodologies. This meant that identifying similarities between studies was often challenging. Despite the use of broad search terms and a systemic search strategy, there is a possibility some studies were also missed in this review.

The findings of this review reinforce how integrating co-design research methodologies can ensure that strategies are grounded in communities’ experiences and needs, in this case, for addressing student FI in the higher education setting. This review uncovered a variety of strategies that researchers, students and university stakeholders can adopt in their respective institutions. Co-design approaches can bring about additional benefits such as enhanced student capacity, improved advocacy efforts, long-term partnerships and a deeper understanding of stakeholders’ priorities and needs. Empowering students to be actively involved in the research and decision-making processes concerning their food environments is critical for achieving equitable, healthy outcomes and long-term systemic changes. The review also identified several barriers associated with utilising such methodologies in higher education settings. As research in this field progresses, future studies should prioritise collaborative co-design approaches when exploring solutions to FI and similar social justice issues affecting students.

## Supporting information

Scutts et al. supplementary material Scutts et al. supplementary material

## Supplementary material

For supplementary material accompanying this paper visit https://doi.org/10.1017/S1368980025000485
